# Effects of Building Direction, Process Parameters and Border Scanning on the Mechanical Properties of Laser Powder Bed Fusion AlSi10Mg

**DOI:** 10.3390/ma17153655

**Published:** 2024-07-24

**Authors:** Juan M. García-Zapata, Belén Torres, Joaquín Rams

**Affiliations:** 1 Departamento de Matemática Aplicada, Ciencia e Ingeniería de Materiales y Tecnología Electrónica, Escuela Superior de Ciencias Experimentales y Tecnología, Universidad Rey Juan Carlos, C/Tulipán s/n, 28933 Madrid, Spain; belen.torres@urjc.es (B.T.); joaquin.rams@urjc.es (J.R.); 2 Instituto de Tecnologías para la Sostenibilidad, Universidad Rey Juan Carlos, C/Tulipán s/n, 28933 Móstoles, Spain

**Keywords:** LPBF, aluminium alloys, microstructure, mechanical testing

## Abstract

The variability arising from the LPBF process, the multitude of manufacturing parameters available, and the intrinsic anisotropy of the process, which causes different mechanical properties in distinct building directions, result in a wide range of variables that must be considered when designing industrial parts. To understand the effect of these variables on the LPBF manufacturing process, the performance of the AlSi10Mg alloy produced through this technique has been tested through several mechanical tests, including hardness, tensile, shear, and fracture toughness. The results have been correlated with the microstructure, together with manufacturing parameters, building directions, border scanning strategy, and layer height. Significant differences were observed for each mechanical behavior depending on the configuration tested. As a result, an anisotropic material model has been developed from tested samples, which allows to numerically model the alloy and is unique in the current literature.

## 1. Introduction

Aluminum alloys find extensive use in the automotive and aerospace industries due to their superior corrosion resistance and high strength-to-weight ratio, which makes them ideal for structural components. They also offer an optimal balance between mechanical performance, light weight, and durability [1,2].

Among all of these, Al–Si alloy possess the best processability, especially at Si contents near the eutectic composition, due to its low melting point and its reduced solidification temperature range, which hinder solidification cracking and make them particularly suitable for welding [3,4]. Moreover, the addition of magnesium in these alloys increases their strength, promoting the formation of M$g_{2}$Si precipitates, which form during aging and act as barriers to dislocation movement under stress [5,6]. This explains the excellent balance between mechanical properties and processability of the AlSi10Mg alloy.

This composition can currently be manufactured using different techniques, which include traditional manufacturing processes, such as casting, forging, extrusion, powder metallurgy, and additive manufacturing (AM). Among the different AM technologies, laser powder bed fusion (LPBF) has arisen as the most dominant, particularly for aluminum alloys, because it has the following strengths: (i) it can produce complex geometries with high dimensional precision and minimum waste, (ii) lightweight objects can be produced by LPBF using topology optimization with almost no restrictions, (iii) machining and other mechanical processes can be avoided [7], and (iv) compared to subtractive methods, this technology reduces energy, waste, cost, and the environmental impact required for parts production, because only the material that constitutes the pieces is melted, leaving the rest ready to be recycled. These strengths increase with the complexity of the pieces, allowing manufacture of geometries impossible to produce by traditional manufacturing methods.

In addition, not only do the complex geometries achievable by this technology present an advantage with respect to traditional production methods, but the material itself has a unique microstructure with special properties. This highly refined microstructure produced by the fast-cooling rates of the LPBF process provides substantial enhancements in hardness and strength. However, these outstanding values for strength and hardness are also accompanied by a decrease in ductility and toughness [8,9,10]. In addition, the properties obtained through the LPBF process depend on the material used and the manufacturing parameters, such as scanning speed, layer height, laser power, and border scanning strategy, as studied by [11].

Commercial additively manufactured parts usually present two regions with different microstructures: the contours and the core. These distinct microstructures are caused by the different scan strategies and process parameters used when scanning these two areas. The core is usually scanned first and the contours afterward in each layer, with a slower scan speed and a higher laser power, which results in a higher energy density [12]. In addition, while the core scanning pattern is shifted layer after layer to avoid overlapping, the contour is scanned following the same path in each layer, which creates a discontinuity in the microstructure, which promotes the appearance of a porosity layer where both zones meet, as observed by Schnabel et al. [13]. Although the rougher surface produced by without border scanning samples is visible at first sight, its effects on the mechanical properties have still not been reported in the literature. Karimi et al. [14] are among the few who investigated the effect of the number of contours on the microstructure of the AlSi10Mg alloy and found significant differences in grain morphology and surface roughness. In addition to these two zones, the top and bottom layers also present a different scanning process, because they are usually remelted to remove the marks left by the scanned hatches, since they are frequently visible if a remelting layer is not used.

The LPBF manufacturing process is also an intrinsically anisotropic technique, since the layered manufacturing process creates a direction that strongly differs from others. Therefore, tensile and shear properties must be determined for each building direction to be able to characterize the alloy and build an orthotropic material model, which allows prediction of its response to mechanical stresses. Tensile characterization is the preferred mechanical characterization in the literature, since it gives a basic insight into mechanical properties, and is easy to perform [15,16,17,18]. On the other hand, shear properties are crucial in designing short beams and bolted joints, but they are difficult to perform and there is almost no literature. The only work on the shear behavior of LPBF AlSi10Mg found in the literature is from Ben Amir et al. [19], who conducted a dynamic shear test with a dynamic punch assembly. In their work, the shear response of a LPBF produced AlSi10Mg to quasi-static and dynamic loads was obtained in different building directions for one specific manufacturing condition. However, the AlSi10Mg alloy has not yet undergone rigorous shear testing to obtain its elastoplastic response to a static load.

Fracture toughness is also an essential property of structural alloys, which defines their damage tolerance and reliability, since it determines the resistance of the material to crack growth. It has been reported to increase, when compared to casting, due to its particular microstructure of fine melt pools. Paul et al. [20] reported values for fracture toughness of 3.7 kJ/mm^2^ and 18.6 MPa$\sqrt{m}$ for a T6 heat-treated as cast AlSi10Mg and ~10 kJ/mm^2^ and ~30 MPa$\sqrt{m}$ for LPBF AlSi10Mg. In addition, Hitzler et al. [21] reported values of 42 MPa$\sqrt{m}$ for the LPBF process compared to the 16–42 MPa$\sqrt{m}$ of wrought aluminum alloys. Yet studies of LPBF produced AlSi10Mg are still scarce, moreover, and vary depending on the powder manufacturer, LPBF machine, and process parameters [22,23].

Consequently, the complex microstructure, the wide variety of manufacturing parameters, and the inherent defects of the parts produced by the LPBF process make them complex to use, especially for transport and industrial purposes where an unexpected failure could have catastrophic consequences. Therefore, to design functional parts, all these factors must be studied under different stress conditions to verify if what is suitable for one stress state is applicable for all, and to analyze each mechanical behavior under the same manufacturing conditions.

In this work, surface hardness, tensile, shear, and fracture toughness properties have been studied through different process parameters, which include the three main building directions, two layer heights, and the use or not of border scanning, reporting significant variations among the different configurations. The obtained tensile and shear modulus, together with plastic curves and the Poisson ratio, have been used to create an orthotropic material model of the LPBF-produced AlSi10Mg. Furthermore, the fracture results obtained also provide the required information to simulate material resistance to crack growth. Therefore, the mechanical behavior of as-built AlSi10Mg is for the first time fully defined.

## 2. Experimental Procedure

### 2.1. Manufacturing Conditions

**Figure 1:**
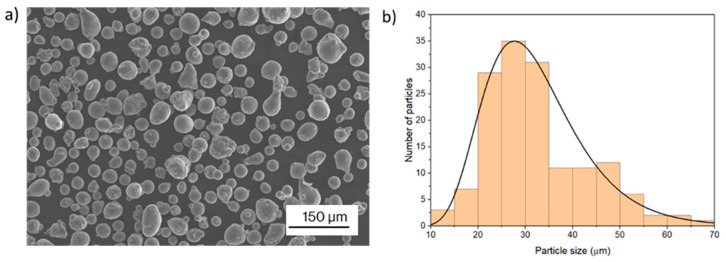
Aluminum powder used: (**a**) SEM images, (**b**) Particle size distribution. The line represents an approximate particle size distribution.

The raw AlSi10Mg powder used was supplied by Renishaw, Wotton-under-Edge, UK. It was composed of spherical particles, as shown in Figure 1a, with a particle size ranging from 20 to 60 µm according to the manufacturer. The particle size distribution was analyzed, measuring the diameter of 200 particles, and an average size of 30 µm was reported, as shown in Figure 1b.

The specimens were produced with a Renishaw AM 400 LPBF machine equipped with a 400 W ytterbium fiber laser. Argon was employed as the inert gas and the temperature of the mounting plate was kept constant at 170 °C to reduce residual stresses and boost the precipitation of Si particles into the Al matrix [24].

For evaluating the effect of manufacturing parameters on mechanical properties, two values of layer height were used, each one with its own manufacturing parameters, as listed in Table 1. In addition, two different laser parameters were used for the border scanning on each layer height (Table 1). The energy density and the build rate obtained for each configuration are also displayed in Table 1, since the energy density, in spite of its limitations [25,26], has proved to be valid parameter to describe the manufacturing conditions and the build rate is an indicator of production capacity. They were calculated according to Equation (1), with v being the scanning speed, h the hatching distance, t the layer height and *P* the laser power W.

(1) $$ED=\frac{P}{v\cdot h\cdot t};BR=v\cdot h\cdot t$$

**Figure 2:**
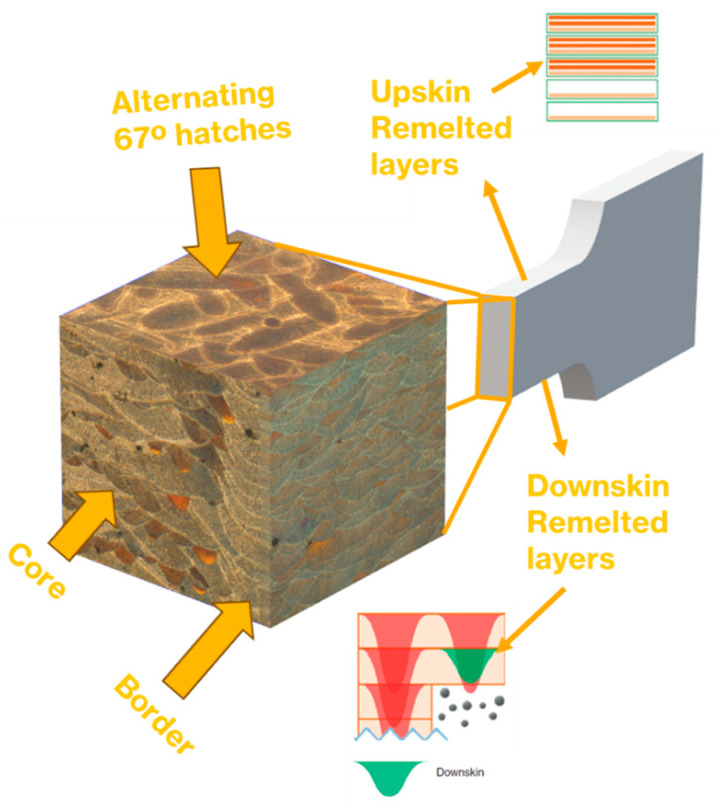
Internal scanning structure of an edge sample; orange and green means remelted layers.

As previously mentioned, the internal microstructure of the samples consists of a core region and a border zone together with the top and bottom remelted layers. The core is where the infill conditions are used and covers the bulk of the sample. This zone is scanned with an alternating angle (Figure 2) in order to eliminate the chance of scan lines repeating themselves directly on top of each other, creating poor material properties. A rotation angle of 67° is usually used, as it will take 180 slices before another will be generated in the same scan direction. On the other hand, the border and the remelted zones are always scanned with the same pattern.

The border zone and the remelted layer locations are shown in a transversal cut of an edge sample (Figure 2). The border zone is scanned with the border conditions, and it is built on the external surfaces. A porosity layer is generated where the core and the border zone converge due to the large disparities in scanning conditions [13]. Finally, remelted layers are generated on the top and bottom surfaces.

**Figure 3:**
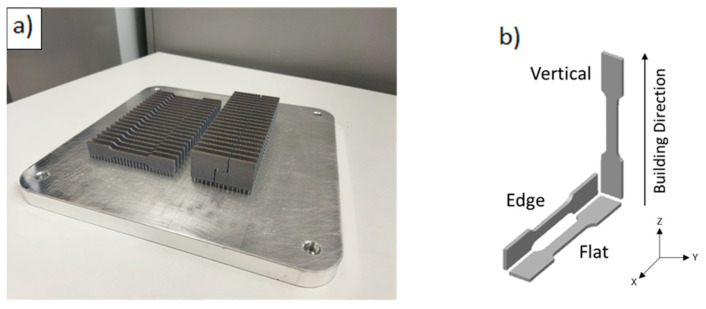
(**a**) Production batch of edge samples; (**b**) building directions.

All parts were produced in batches, as shown in Figure 3a, using coarse cylindric support structures with a diameter of 1 mm on the platform and 0.6 mm on the parts. Support spacing was inferior to 1 mm to ensure a correct heat transmission between the parts and the substrate and to avoid crack initiation on the unsupported zone.

**Figure 4:**
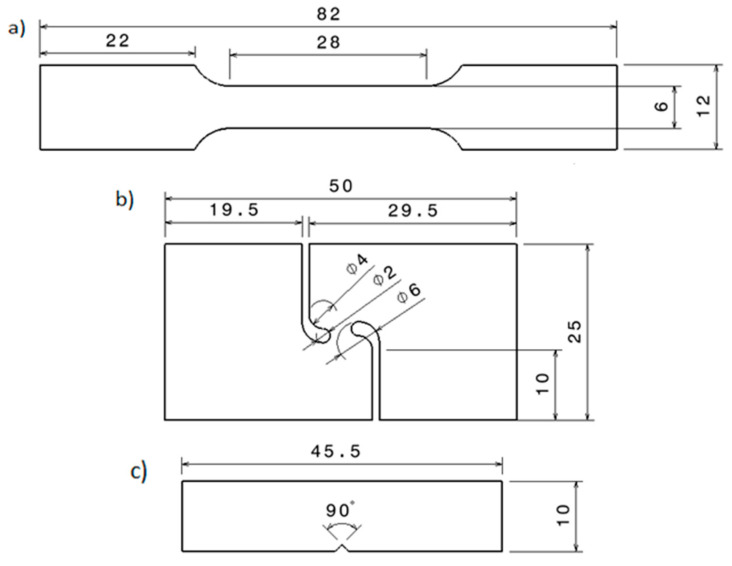
Specimen geometries: (**a**) tensile specimen (**b**) shear specimen (**c**) fracture specimen.

Parts in three different building directions were produced to evaluate the material properties in the three principal planes, which are defined in Figure 3b. The coordinate system defined by the ISO 52921 [27] standard for additive manufacturing terminology was followed to establish each orientation. It defines the *Z* axis as the building direction and the *Y* and *X* axes as the parallel and perpendicular directions to the front of the machine. It also defines the building directions depending on where the sample is located on the XY, XZ and ZY planes. For simplicity, the XY, XZ and ZY building directions defined by the standard were renamed as flat, edge, and vertical. The different geometries manufactured are shown in Figure 4. They correspond with tensile (Figure 4a), shear (Figure 4b), and fracture (Figure 4c) samples. The whole experimental process is detailed in Table 2, where a total of 19 conditions were analyzed through tensile, shear and fracture tests; three to five samples were used for each configuration.

### 2.2. Surface Hardness

Surface hardness was evaluated to determine the impact of manufacturing parameters, building directions, and border scanning on the surface. Vickers microhardness tests were conducted employing a 500 g load for a 10 s dwell time. The hardness testing device used was an “Innovatest 500” (Maastricht, The Netherlands), and ten indentations were made across the length in areas of the specimen with sufficient distance to the edges and nearby indentations. The specimens were smoothed using 1200-grit sandpaper to homogenize the surface and eliminate the rough outer layer resulting from the LPBF process.

### 2.3. Tensile Testing

Tensile samples were designed with customs dimensions based on ASTM E8 [28] Figure 4a. For tensile tests, a universal testing machine (Zwick/Roell, Ulm, Germany) equipped with a load cell of 100 kN was used to record the force. The crosshead speed used was 1 mm/min, and strains were measured by digital image correlation (DIC) with a virtual extensometer (GOM correlate).

### 2.4. Shear Testing

The specimen used for shear tests was adopted from [29] and it is shown in Figure 4b. This specimen geometry is relatively simple to fabricate, does not require through-thickness machining, ensures low and stable values of stress triaxiality on the shear zone, and can be tested with a universal testing machine without the necessity of additional fixtures. Compared to that proposed by ASTM B831 [30] standard, the outer internal radius of 3 mm, the wider zone subjected to tensile stresses, and the asymmetry of the notches have been reported to reproduce shear conditions more accurately and prevent the specimens from failing on the notches instead of on the shear zone.

**Figure 5:**
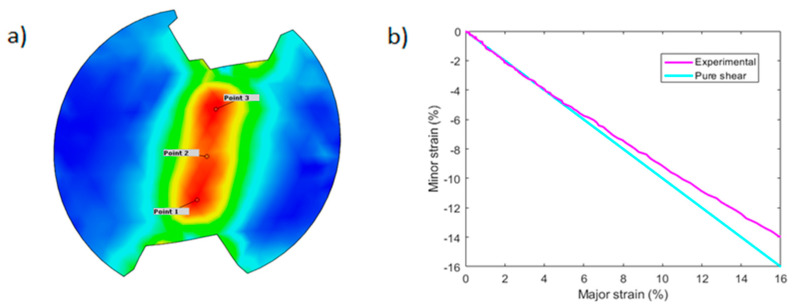
Shear testing evaluation procedure: (**a**) measurement points, (**b**) minor vs major strain values. Colors correspond with different values of strain.

The testing machine was the same as that used for tensile tests, but in this case, it was equipped with a 5 kN load cell, and a crosshead speed of 0.5 mm/min was used instead, as displacements are very limited in shear testing. Shear angles were recorded in the shear zone by applying digital image correlation (DIC) to the entire surface. Three points in the zones with higher strain values were tracked during the test to evaluate shear conditions. Shear strains were evaluated on these points, measuring both principal strains, and the scheme used is shown in Figure 5a. For simple and pure shear deformations, to satisfy conditions for both plane stress and plane strain loading, there must be no hydrostatic stresses, so the individual normal strains may be non-zero, but they must sum to zero [31]. Simple shear behavior was observed on most specimens, particularly on the elastic zone, and deviated in the plastic zone when subjected to high strains, as shown in Figure 5b.

### 2.5. Fracture Testing

Fracture testing was performed with the same machine and conditions used for shear testing. The specimen utilized was a Single Edge Notched Bending (SENB) in three-point bending (Figure 4c). An initial crack of 2.5 mm was made with a 0.25 mm thick cutting disk, *a*/*W* = 0.65 and *W*/*B* = 2, according to ASTM E1820 [32]. The thickness of the specimens was set to 5 mm to keep most of the specimens in plane strain conditions and ensure a high level of triaxiality on the crack plane. Although thicker specimens could have been produced, the minimum possible thickness was chosen to facilitate productivity. Crack mouth opening displacement (CMOD) was also evaluated with DIC to compare force-CMOD curves among the different configurations.

### 2.6. Microstructure of the Manufactured Samples

Microstructures were characterized on the cross-section of tensile samples using an Optical Microscope DMR (Leica, Wetzlar, Germany) equipped with a CCD camera model DFC 320, and a Scanning Electron Microscope Philips XL-30 ESEM (Eindhoven, The Netherlands) equipped with an Energy Dispersive X-ray Spectrometer (EDAX ultra-thin window (UTW) EDS detector). Samples were mechanically polished (up to 1 mm) and etched in Keller’s solution to reveal the microstructure.

## 3. Experimental Results

### 3.1. Microstructural Analysis

The LPBF manufacturing process of melting material layer after layer together with the fast and directional cooling rates create a unique microstructure composed of melt pools arranged according to the scanning pattern and the manufacturing parameters. Moreover, depending on the building direction, the melt pools will be located in different zones and oriented one way or another.

**Figure 6:**
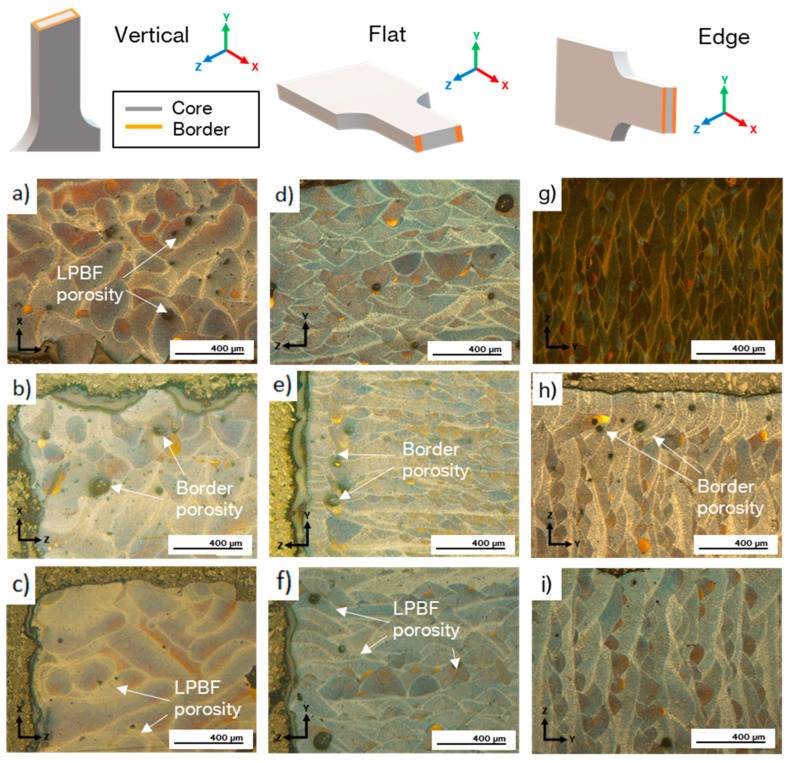
OM micrographs of the different building directions produced at 60 µm: (**a**) vertical 60 µm microstructure, (**b**) vertical 60 µm, (**c**) vertical 60 µm WB, (**d**) flat 60 µm microstructure, (**e**) flat 60 µm, (**f**) flat 60 µm WB, (**g**) edge 60 µm microstructure, (**h**) edge 60 µm and (**i**) edge 60 µm WB.

The as-built microstructures are shown in Figure 6 for each building direction. The first row corresponds to the core as-built microstructures, the second to the border zones with border scanning, and the last to the border zone without border scanning samples. Vertical samples are depicted in the first column, flat samples in the second, and edge samples in the third. The location of the border scanned zone for each building direction is represented in the scheme of Figure 6, together with the coordinate system of each cross section. The border is scanned on the top and bottom surfaces of edge samples, on the lateral surfaces of flat samples and on the whole perimeter of vertical samples.

Porosity is observed in all samples but as observed in Figure 6, it can be divided in two types depending on cause. The first type of porosity is produced by the manufacturing process, due to solidification defects, and it is present throughout the microstructure, primarily on the boundaries of melt pools on flat and edge samples. It was also frequently located inside the melt pools in vertical samples. The second type is the border porosity, which is caused by the different solidification conditions between the core and the border zone due to the different energy density used on each zone. It is present on the boundary between the core and the border of edge and flat samples with border scanning.

Regarding the different arrangements of melt pools and their shape, edge and flat samples presented elliptical melt pools grouped in rows with some degree of overlapping, whereas vertical samples presented elongated melt pools randomly distributed. This is because this is the only orientation in which the laser scans in the surface of the cross section and volume hatches are observed.

**Figure 7:**
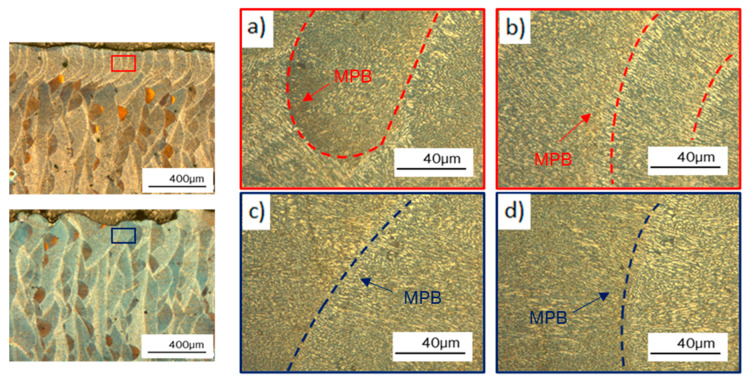
OM micrograph of edge samples with and without border scanning: (**a**,**b**) edge 60 µm, (**c**,**d**) edge 60 µm WB; color boxes mark the observation zone.

The border scanned zone was analyzed on edge samples to investigate the effect of the border scanning on the microstructure. Figure 7a,b illustrates the microstructure of edge samples with border scanning, whereas Figure 7c,d shows the microstructure without border scanning samples. Melt pool boundaries (MPBs) are marked with dotted lines. A clear difference between grains was not observed in both border scanning strategies. However, more MPBs were observed on samples with border scanning, with fine elongated grains oriented in the solidification direction, than on samples without border scanning. In contrast, the MPBs were more differentiated and less frequent in samples without border scanning.

**Figure 8:**
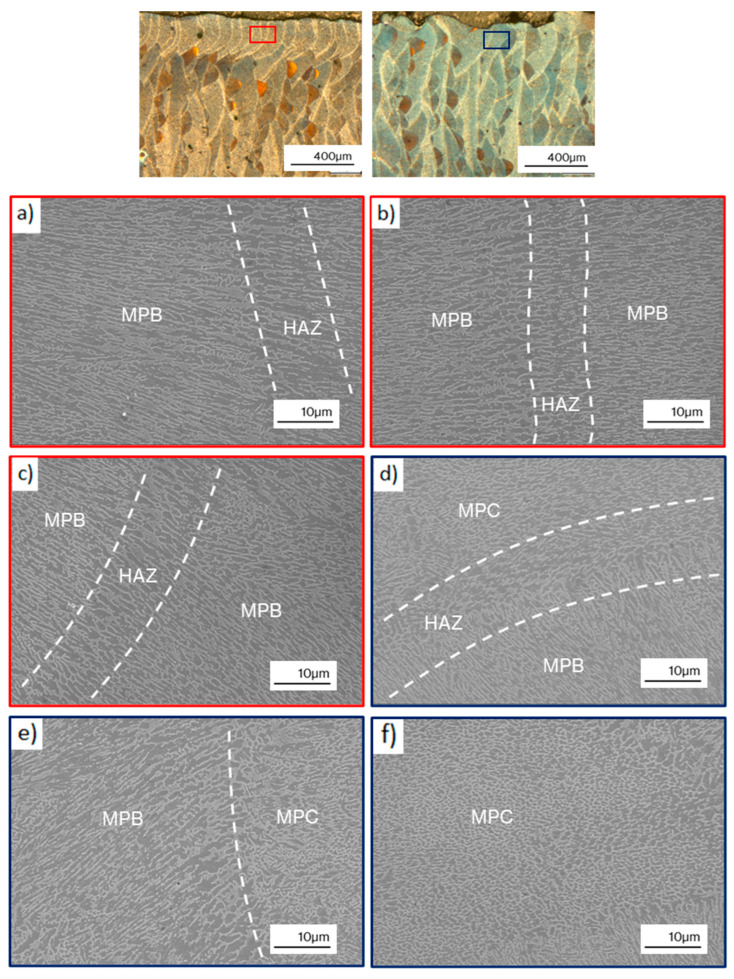
SEM micrographs for both border scanning strategies at 60 µm: (**a**–**c**) edge 60 µm, (**d**–**f**) edge 60 µm WB; color boxes mark the observation zone.

Additional SEM micrographs of edge samples with border scanning are shown in Figure 8a–c, and the microstructure of samples without border scanning in Figure 8d–f. The macro-scale melt pools are visible because of the morphological transition between the melt pool center (MPC), the MPB, and the heat-affected zone (HAZ). The MPC consists of an ultra-fine α-Al matrix and a Si-rich eutectic network with a cellular morphology as a secondary phase, while the MPB exhibits a coarser microstructure of α-Al cells mixed with columnar dendrites of the Si-rich eutectic phase. The dendrite spacing on the MPC was around 0.5 µm while on the MPB zones it was around 1 µm. Such microstructural features correspond well with the previous results observed on the LPBF AlSi10Mg [15]. The HAZ showed a partial disintegration and spheroidization of the Si-rich eutectic network due to the high temperatures reached on this zone.

The MPB zones were frequently observed on the exterior surfaces of border scanned samples presenting oriented columnar dendrites mixed with the HAZ zones. However, the MPC zones with fine cellular morphology were not observed with the same frequency than in without border scanning samples and were located only in particular areas. This can be attributed to the different overlapping between melt pools for each border scanning strategy. Border scanned samples presented more overlapped melt pools as shown in Figure 6h, in contrast to without border scanning samples, where the melt pools were more detached with little overlapping among them. Therefore, as the melt pools are more overlapped, the more frequent the MPB zones become, leaving the MPC in more localized areas.

### 3.2. Hardness Analysis

**Figure 9:**
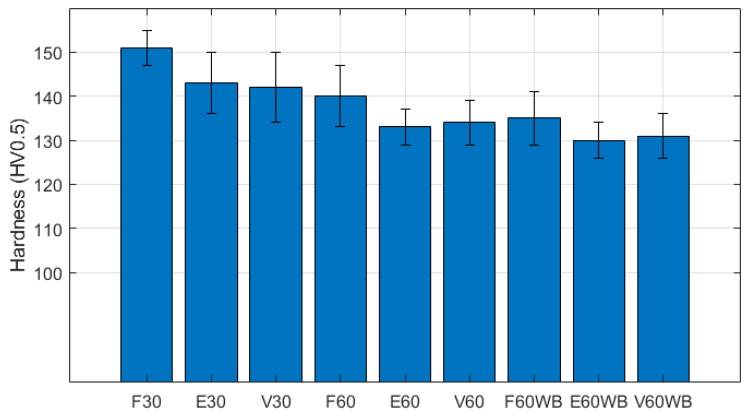
Hardness values of tested specimens at different process parameters, building directions, and border scanning.

The surface hardness values measured are collected in Figure 9 for the different conditions tested. The higher hardness values corresponded to flat specimens, which stood out with values which were ~10 HV higher than in edge and vertical samples. This distinct hardness values may have been due to the presence of the top and bottom remelted layers, which only coincide with the indented surfaces on flat samples. Hence, remelted layers seem to have an impact on hardness. Furthermore, samples produced with a layer height of 30 µm presented higher values than those produced with 60 µm, which again implies more melted layers on the same depth. Therefore, there seems to be a correlation between remelted layers and hardness.

The samples which were manufactured without border scanning showed inferior hardness values for all building directions and layer height than those with border scanning, with values that were ~5 HV (3%) lower. This can be explained by means of the bigger grain size present on the surface of without border scanning samples that arises from the higher energy used in the perimetral laser scanning.

Other authors have reported inferior values of surface hardness [33,34], which can be attributed to differences in the production process, such as the temperature of the mounting plate, the laser power, and the scanning speed. Praneeth et al. [33] reported hardness values ranging from 107 to 123 HV depending on the laser parameters used, obtaining the higher values with a scanning speed of 1000 mm/s and 250 W laser power. In addition, Serjouei et al. [34] obtained hardness values of 125 HV with a scanning speed of 1300 mm/s and a laser power of 370 W. Hardness values are related to the cooling rate because higher cooling rates result in finer grains that provide increased surface hardness. Furthermore, an appropriate temperature of the mounting plate boosts the precipitation of Si particles, which also increase surface hardness [15]. In this work a faster scanning speed was used (1600 mm/s) together with a higher laser power (400 W), and the mounting plate temperature was kept constant at 170 °C during the whole manufacturing process. The high cooling rate produced, together with the temperature of the mounting plate used, provided hardness values of up to 150 HV.

### 3.3. Tensile Analysis

**Figure 10:**
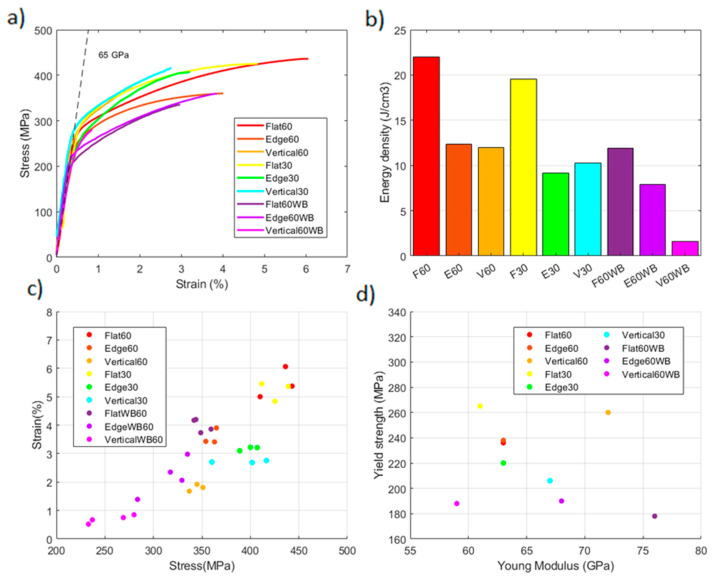
Tensile testing results for each configuration: (**a**) stress–strain curves, (**b**) energy density, (**c**) max stress–strain, (**d**) elastic properties.

The stress–strain curves of the LPBF AlSi10Mg are shown in Figure 10a. All the stress–strain curves displayed a similar shape, where the applied stress reached a maximum value, and then abruptly failed during the strain hardening stage without necking. The curves adjusted well to a bilinear hardening law with an average modulus of 63 GPa and a tangent modulus of 3.5 GPa. Two different manufacturing conditions were tested for 30 µm and 60 µm layer height. Each manufacturing condition was also evaluated in the principal building directions, with and without border scanning, as listed in Table 2. Even though building directions and the presence of border scanning do not affect the solidification process directly, they have an influence on mechanical properties as they affect the stress propagation through the microstructure, so their effect was also analyzed.

The energy density absorbed during tensile tests, calculated as the area beneath the stress–strain curves, is plotted in Figure 10b. Flat specimens presented the highest values with 22 J/cm^3^, followed by edge and vertical samples with values around 12 J/cm^3^ for the 60 µm samples. The 30 µm samples absorbed less energy than the 60 µm samples with a decrease of around 2 J/cm^3^ for each building direction. The samples produced without border scanning also absorbed less energy than those with border scanning, especially flat (12 J/cm^3^) and vertical (2 J/cm^3^) samples.

The measured values of tensile strength and strain to failure are shown in Figure 10c. A wide range of resistance and ductility values were obtained, ranging from 429 MPa and 5.71% strain for flat 60 µm samples to 254 MPa and 0.7% strain for 60 µm vertical samples without border scanning. The results showed that building directions, together with the presence of border scanning, have a significant effect on ductility and resistance, while manufacturing parameters do not have a significant role on tensile properties.

Regarding the building directions, flat specimens showed the highest values of resistance with 429 MPa, followed by edge samples with 360 Mpa, and lastly by vertical specimens with 344 MPa, for the 60 µm process parameters. For the 30 µm process parameters, flat specimens again reported the higher values of strength with 425 Mpa, followed by edge and vertical samples, in this case with similar values of around 390 MPa. Respecting ductility, flat samples also showed the higher values with 5.7% and 5.2% strain to failure for 60 µm and 30 µm process parameters, respectively. These were followed by edge specimens with values of 3.5% and 3.2% strain to failure and lastly by vertical samples with values of 1.8% and 2.7% strain to failure.

The specimens without border scanning produced at 60 µm presented inferior values for strength and ductility than those with border scanning, with resistance values of 357 MPa, 316 MPa, 254 MPa and 4%, 2.18%, 0.7% strain to failure for flat, edge and vertical samples, respectively. A linear tendency was observed for strength and ductility with building directions and border scanning, which suggests that the tensile response is influenced more by the melt pools arrangement than by the process parameters.

The elastic properties of tensile tested samples are represented in Figure 10d. There were significant differences among building directions, process parameters and border scanning on stiffness and yield strength. Specimens without border scanning presented inferior values of yield strength, which were below 200 MPa, and different values of stiffness depending on the building directions, with flat samples reporting the higher values and vertical samples the inferior. The opposite was observed on samples with border scanning where vertical samples reported the higher values of stiffness with flat and edge samples presenting similar values. Yield strength was between 200 and 265 MPa on these samples. Regarding the process parameters, 60 µm samples reported higher values of yield strength except for flat samples, which showed the opposite tendency. Tensile testing results are listed in Table 3.

**Figure 11:**
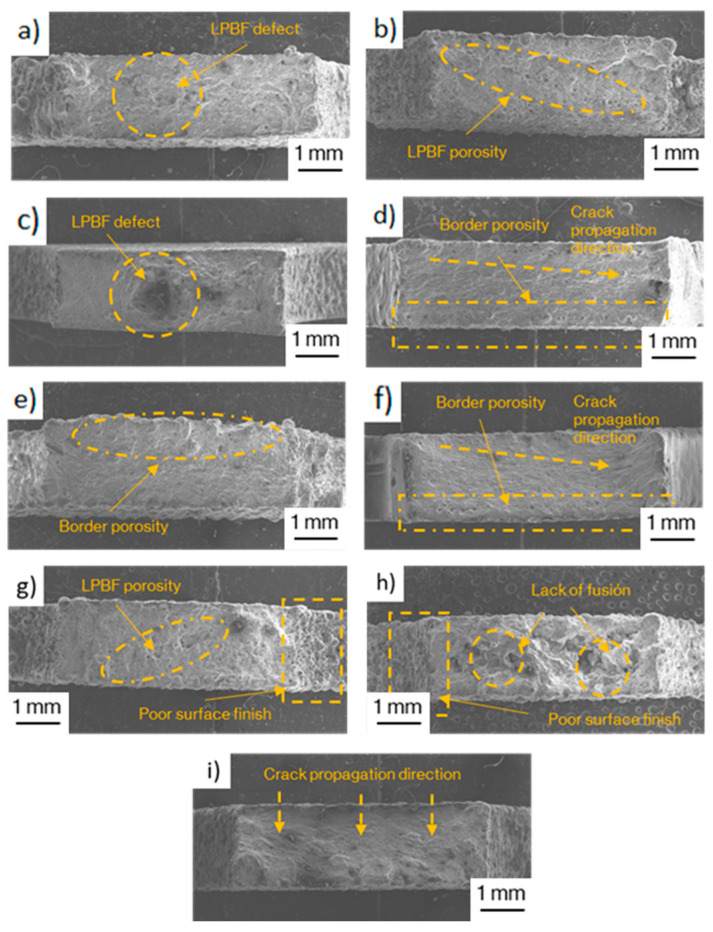
Fracture surfaces of tensile tested samples: (**a**) flat 60 µm, (**b**) flat 60 µm WB, (**c**) flat 30 µm, (**d**) edge 60 µm, (**e**) edge 60 µm WB, (**f**) edge 30 µm, (**g**) vertical 60 µm, (**h**) vertical 60 µm WB, and (**i**) vertical 30 µm.

Fracture surfaces are depicted in Figure 11. Flat specimens (Figure 11a–c) presented a rough surface, together with porosity and defects produced by the manufacturing process. On the other hand, edge specimens (Figure 11d–f) presented a smoother fracture surface and border scanning-induced porosity was observed on the transition zone between the core and the border, but was not observed on without border scanning samples. Vertical specimens (Figure 11g–i) presented a poor surface finish on the lateral surfaces except for 30 µm samples and this was more pronounced on without border scanning samples. In addition, the latter presented a higher porosity than in the other building directions prior to testing, produced by the manufacturing process. The majority of the samples exhibited an oblique fracture, where failure started on an outer surface and propagated until arriving at the other, as depicted in Figure 11i. From a more global perspective, failure initiated at the top and bottom surfaces of vertical samples, while it started at the inner zone on flat samples. In edge samples, failure started at the lateral surfaces where they were connected to the substrate by the support structures. This indicates that surface finish played a more determinant role in edge and vertical samples while. on flat samples, fracture was initiated by internal defects, as shown in Figure 11b,c.

**Figure 12:**
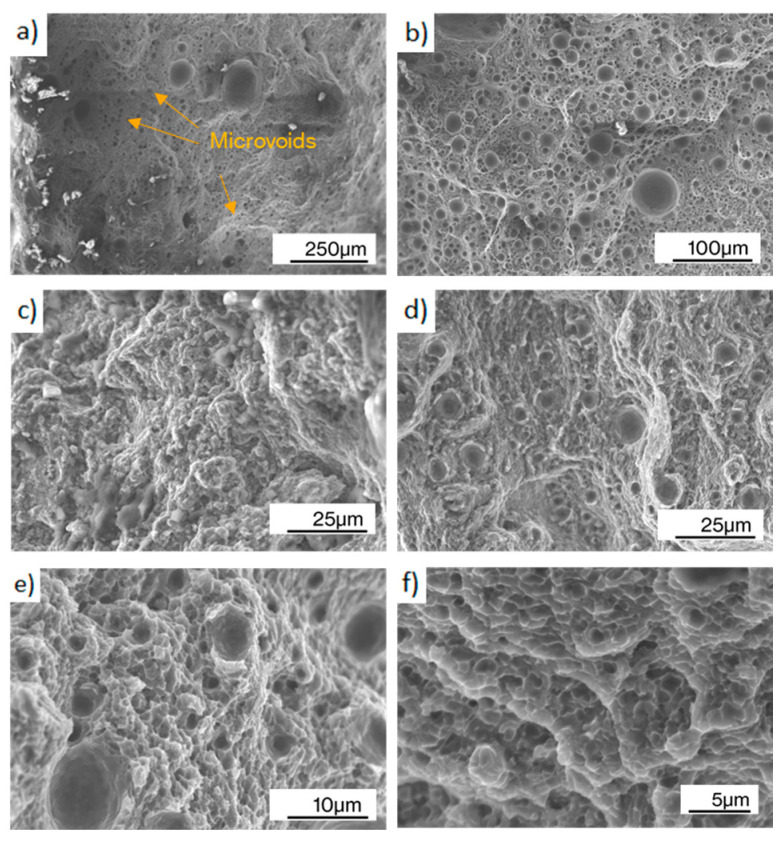
Fracture surfaces of different configurations at various magnifications: (**a**) flat 60 µm, (**b**) vertical 60 µm, (**c**) flat 60 µm, (**d**) edge 60 µm, (**e**) flat 60 µm, and (**f**) flat 60 µm.

Detailed fractography, shown in Figure 12a,b, revealed the presence of micro-voids combined with even and uneven regions on vertical specimens, which is a characteristic feature of mixed brittle-ductile failure. The pores were observed on all magnifications with sizes ranging from 25–50 µm for the bigger ones to 2–10 µm for the micrometric pores, produced by gas porosity and void coalescence. In flat and edge specimens, porosity was also present, but of a larger size than in vertical samples, results that agree with another author’s research [20]. Further fractography at higher magnification (Figure 12c–f) showed a morphology similar to the cellular structure observed in Figure 8, which indicates that the fracture occurred along its boundaries in an intergranular crack propagation. The dimple features presented dimensions comparable to the cells and heat affected zones, as observed by [20,35], which suggests that the Si-rich eutectic restrict dislocation movement within the α-Al matrix, thereby strengthening the material. However, under increasing stress, plastic deformation occurs within the α-Al matrix, leading to the nucleation of cavities, which can subsequently be observed as dimple features at the interface between the Si-rich eutectic and the α-Al matrix.

### 3.4. Shear Analysis

**Figure 13:**
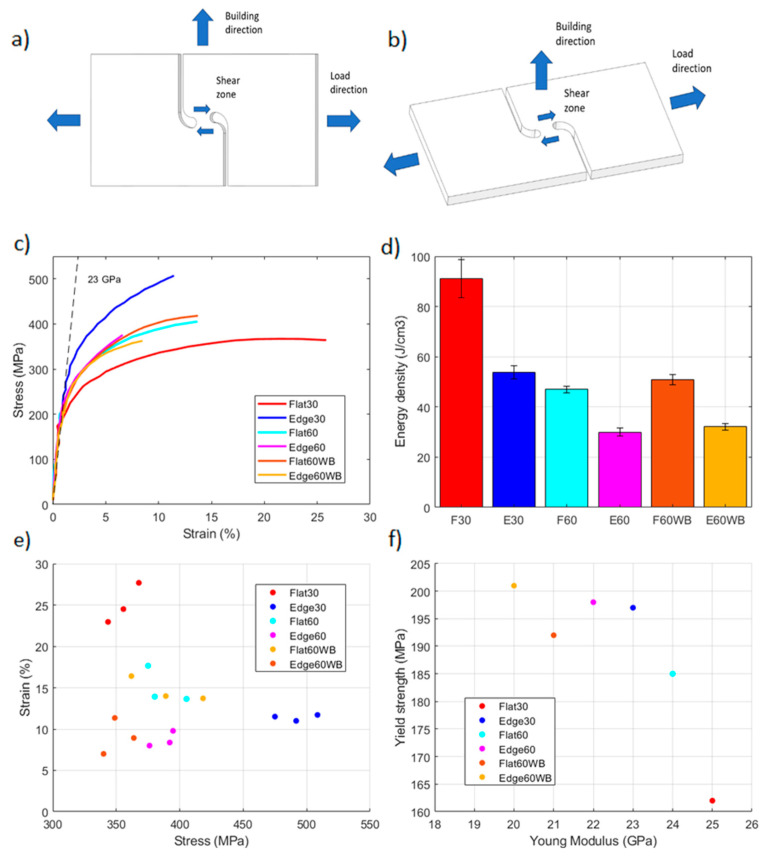
Shear testing results and configurations: (**a**) edge building direction (**b**) flat building direction, (**c**) shear stress–strain curves, (**d**) energy density, (**e**) strength and ductility, (**f**) elastic properties.

As mentioned earlier, LPBF materials are anisotropic so, depending on the building direction the stress will propagate through or across layers. Shear stress propagates across layers in edge samples, whereas it propagates through layers on flat samples, as shown in Figure 13a,b. Averaged shear stress–strain curves are presented in Figure 13c for the six configurations tested, the energy density absorbed in Figure 13d, the strength and ductility values in Figure 13e and the elastic properties in Figure 13f.

The higher values of energy density were obtained by flat specimens, with the 30 µm samples reporting an average energy density of 80 J/cm^3^ and flat 60 µm samples 45 J/cm^3^. Edge specimens reported values of 48 J/cm^3^ and 27 J/cm^3^, respectively, for the 30 µm and 60 µm process parameters. In this case, with and without border scanning samples reported similar energy density values, being slightly higher on samples without border scanning.

The effect of process parameters was significant in this case, as edge 30 µm specimens showed a 20% increase in strength compared to 60 µm samples, while flat 30 µm specimens, in contrast, showed an increase in ductility of 50% compared to 60 µm samples. Values for strength and ductility of 355 MPa and 24% were obtained for flat 30 µm samples and 491 MPa and 11% for edge 30 µm specimens.

Building directions in this case, did not affect strength for the 60 µm process parameters (387 MPa), but an important increase in ductility was obtained by flat samples with a 15% strain to failure, in comparison with the 9% obtained by edge samples. Regarding the effect of the border scanning, specimens without border scanning presented similar values of strength and ductility, in contrast to tensile behavior, where they presented inferior values. In this case, only the strength of edge samples was reduced, obtaining values of 350 MPa.

The average shear modulus was around 23 GPa considering only specimens with border scanning. An influence of building directions, process parameters and border scanning was observed on stiffness. Flat specimens presented the highest values for stiffness with 25 GPa and inferior values for yield strength, with 169 MPa for 30 µm samples, and 24 GPa modulus and 185 MPa yield strength for 60 µm. Edge samples presented inferior values for stiffness but higher values for yield strength, with 23 GPa and 197 MPa for 30 µm samples, and 22 GPa and 198 MPa for 60 µm. Samples without border scanning presented inferior values for stiffness with 21 GPa and 20 GPa, and higher values for yield strength with 192 and 201 MPa for 60 µm flat and edge samples, respectively. All results are summarized in Table 4 for all building directions and process parameters.

**Figure 14:**
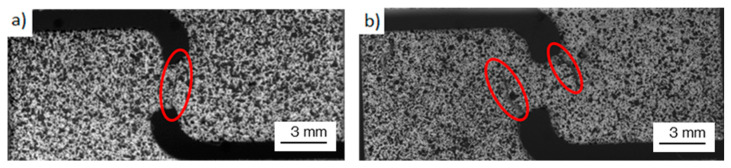
Shear test fracture surfaces: (**a**) Shear zone fracture of flat 60 µm, (**b**) Notch fracture of edge 60 µm; red circles mark the zone of fracture initiation.

**Figure 15:**
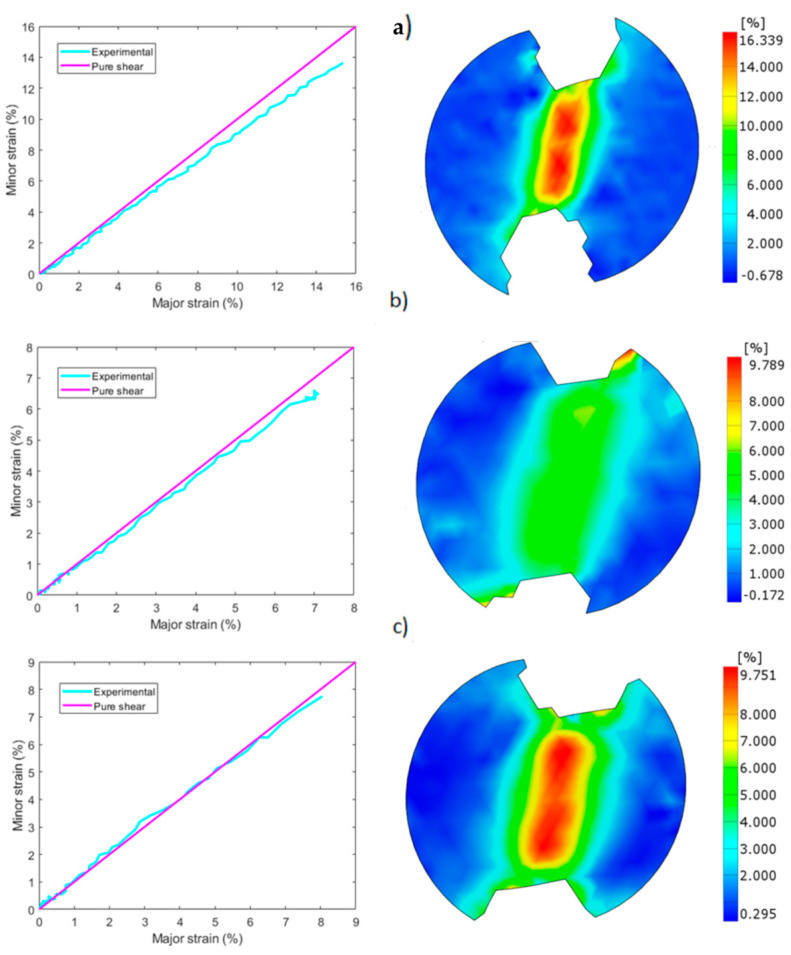
Major/minor shear strain and shear strain distributions: (**a**) flat 30 µm, (**b**) edge 30 µm, (**c**) flat 60 µm.

**Figure 16:**
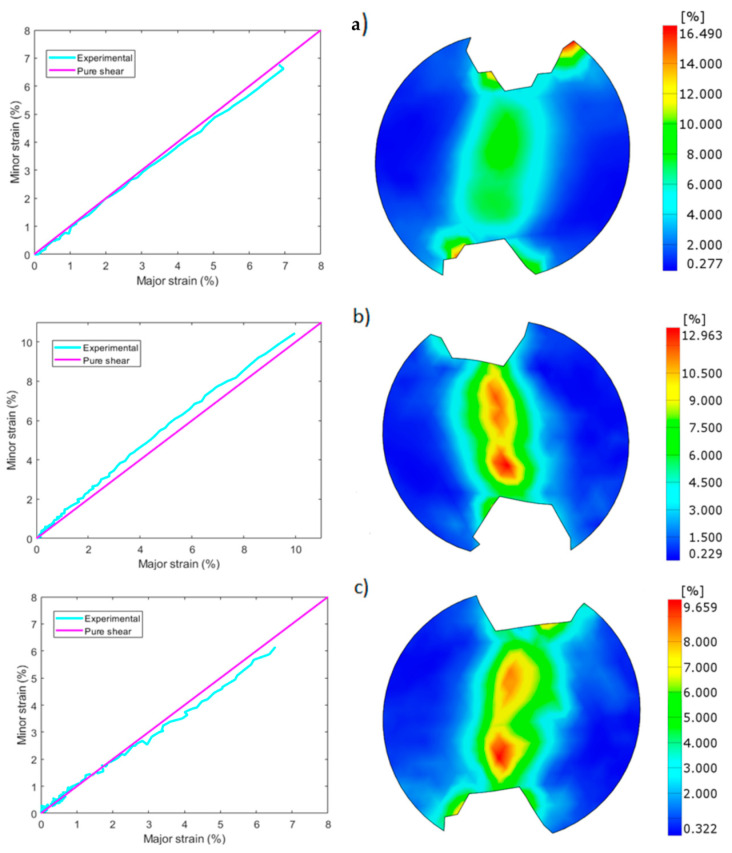
Major/minor shear strain and shear strain distributions: (**a**) edge 60 µm, (**b**) flat 60 µm, WB (**c**) edge 60 µm WB.

Fracture surfaces are shown in Figure 14, where two different modes of failure were observed. In the first, Figure 14a, failure occurred on the shear zone with little damage on the notches, proving that the specimen failed due to shear and that tensile stresses had little influence. On the second, failure occurred at the notches due to tensile stresses, far away from the shear zone (Figure 14b). The strain state of the shear zone prior to failure was analyzed with DIC to compare the shear behavior and the strain distributions of the different configurations, where flat 30 µm (a), edge 30 µm (b) and flat 60 µm configurations are shown in Figure 15, and edge 60 µm (a), flat 60 µm WB, (b) and edge 60 µm WB (c) in Figure 16.

All specimens presented values close to simple shear behavior in the elastic zone but, as the specimen began to deform plastically, major strains started to exceed minor strains and tensile stresses started to prevail, except in flat 60 µm samples without border scanning (Figure 16b), where the opposite was observed, compressive stresses prevailing over tensile.

A remarkable difference in strain concentration was observed at the notches between flat and edge building directions. Whereas edge samples reported inferior values of ductility on the measurement points (6.5%) in comparison to flat samples (14%) for the 30 µm process parameters, both presented similar values of strain at the notches (9.7%), as depicted in Figure 15a,b. Something similar was observed for the 60 µm process parameters. In this case, both building directions reported values of strain between 7 and 9% in the measurement points, but edge samples (Figure 15c) reported much higher values of strain at the notches (16.5%) in contrast to flat samples (7.5%) (Figure 16a). Specimens without border scanning again presented a similar behavior in flat (Figure 16b) and edge samples, Figure 16b reporting values of 10% and 6% strain at the measurement points and values of 3.5% and 7% strain for the notches. This explains the higher tendency of edge samples to fail at the notches, while flat samples mostly failed on the shear zone.

A homogeneous distribution of strains was observed on samples with border scanning, in contrast to samples without border scanning. While the former presented constant values of strain in the shear zone, the latter reported an inhomogeneous distribution of strains, with two zones of high strains close to the upper and bottom notches separated by a low strain zone in the middle of the shear zone.

### 3.5. Fracture Testing

The stress intensity factor (SIF) is commonly used in the literature as a fracture toughness parameter for LPBF AlSi10Mg [36]. However, the specimen size requirement for a valid K_1c_ is not fulfilled by the fabricated specimens, as their thickness is less than that which the ASTM E399 standard [37] specifies for a valid test. This is because this fracture parameter is very limited for ductile materials, since it requires a mostly elastic response to be considered as a valid parameter, which is rarely found on metals. Conversely, j-integral testing described in the ASTM E1820 standard [32] did achieve the minimum thickness required for a valid j-integral test, as shown in Equation (2). This parameter is less restrictive to size requirements, and more appropriate to metal testing, because it is an elastoplastic fracture toughness parameter that considers the energy absorbed by the material prior to failure, in contrast to SIF, which only considers the maximum stress values reached.

(2) $$5\;mm<25\;mm=b>2.5{\left(\frac{K_{Q}}{\sigma_{ys}}\right)}^{2}5\;mm>4\;mm=b>\frac{10J_{max}}{\sigma_{ys}}$$

Although the stress intensity factor (SIF) cannot effectively describe the stress state of the crack, it will be used in addition to the j-integral to report the stress levels reached at the crack. Apart from these parameters, the CMOD has also been accepted by the ASTM E1820 standard [32] as a valid fracture toughness parameter and will also be used to compare the different configurations.

**Figure 17:**
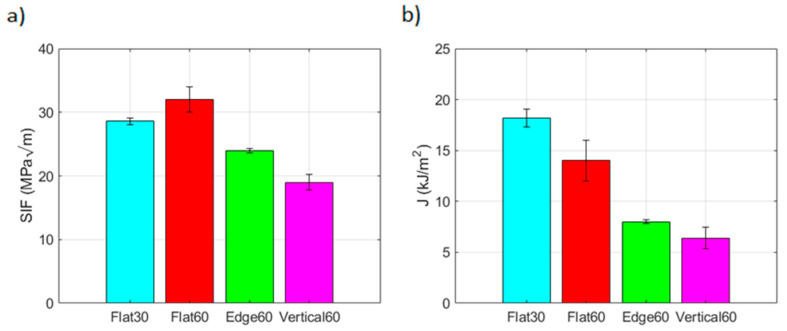
SIF and J values of fracture tested samples. Colors are used for the force-CMOD curves for each configuration. They aim to foster a faster relation between subfigures.

The maximum SIF and j-integral values reached for each configuration tested are depicted in Figure 17a,b. In this case, the effect of the border scanning was not studied as it was not considered relevant, because borders do not affect the stress state of the crack, and only flat samples were tested at 30 µm to simplify the testing procedure.

Significant variations in fracture toughness were observed depending on building directions. Flat specimens showed the higher values of SIF and J-integral (32 MPa$\sqrt{m}$, 14 kJ/m^2^), followed by edge samples (24 MPa$\sqrt{m}$, 8 kJ/m^2^) and lastly by verticals (19 MPa$\sqrt{m}$, 6.4 kJ/m^2^) for the 60 µm layer height. Process parameters also proved to have an impact on fracture toughness. Flat specimens built with a layer height of 30 µm reached a lower SIF (28.6 MPa$\sqrt{m}$) than 60 µm samples. Conversely, they absorbed more energy than 60 µm samples (18.2 kJ/m^2^).

**Figure 18:**
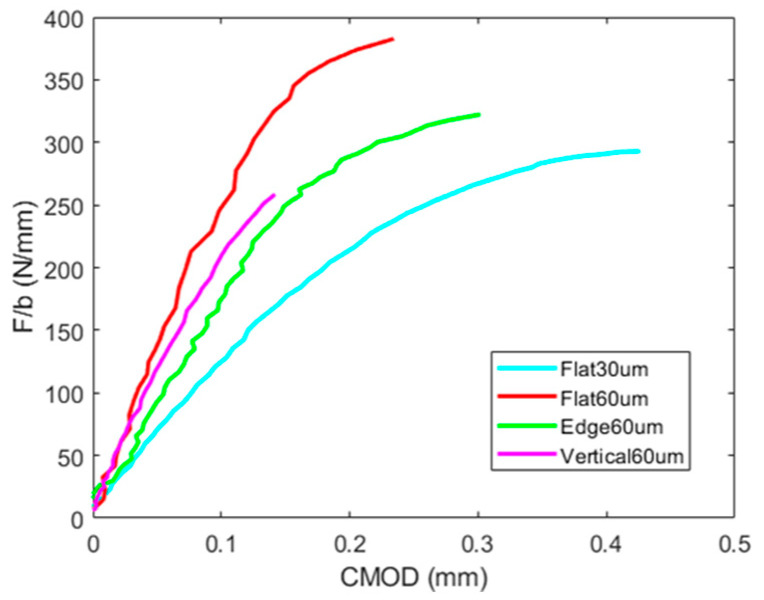
Normalized force vs CMOD of fracture tested samples.

Force-crack mouth opening curves are shown in Figure 18, which have been standardized with the width of the specimens to be able to compare different cracks. Process parameters and building directions proved again to have a significant effect in load-CMOD curves. Flat specimens produced at 60 µm layer height presented significant differences compared with those produced at 30 µm layer height, since they resisted 30% more and deformed 60% less, which is consistent with the SIF and j-integral values obtained. Edge 60 µm specimens presented values between both, and verticals samples showed inferior values for force and CMOD, failing at a much lower deformation.

**Figure 19:**
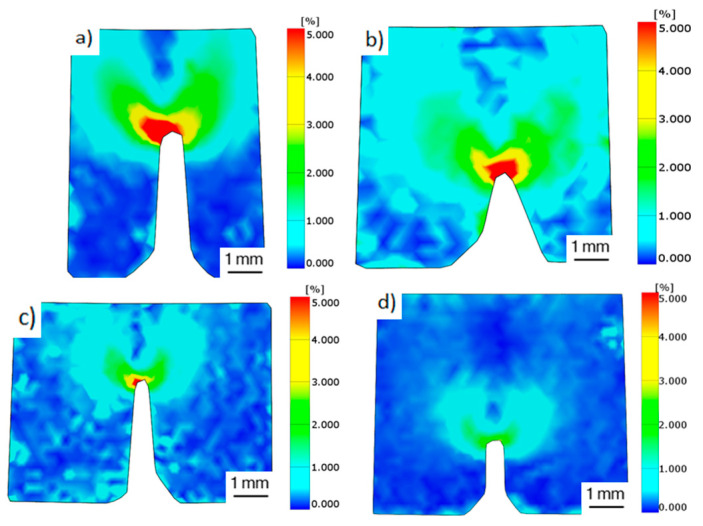
Strain state before failure of fracture tested samples: (**a**) flat 30 µm (**b**) flat 60 µm (**c**) edge 60 µm (**d**) vertical 60 µm.

The stress state present on the crack was essentially plane strain, as shear lips were only present in a small portion of fracture surfaces, close to the external surfaces. The plane stress strain state of the external surfaces prior to failure was recorded with DIC, to compare the behaviour of each configuration, and is depicted in Figure 19. The bigger plastic zones were recorded on flat samples, where strain values over 5% were observed at 1 mm distance from the crack tip, as shown in Figure 19a,b. The size of the plastic zone decreased on edge samples and barely reached a 5% strain at the crack tip (Figure 19c). Vertical samples did not reach the 5% strain value and reported a maximum value of 3% strain at the crack tip (Figure 19d). Although the strain state recorded on the external surface does not represent the strain state inside the crack, because strain values are higher for plane stress, this strain state is proportional to the plane strain state, so it can be useful to compare the differences in the plastic zone size among the different configurations. The plastic zone size for plane stress probed to be closely related with j-integral values reporting similar results, but it was not consistent with SIF values, where flat 60 µm samples reported higher SIF values than 30 µm samples with a smaller plastic zone.

A significant anisotropy was observed in fracture behaviour, where flat samples reported the higher values for fracture toughness and the bigger plastic zones, followed by edge samples and lastly by vertical samples. The higher fracture toughness obtained for flat samples in comparison with edge and vertical samples can be attributed again to the shape and orientation of melt pools, which favour transgranular or intergranular failure.

**Figure 20:**
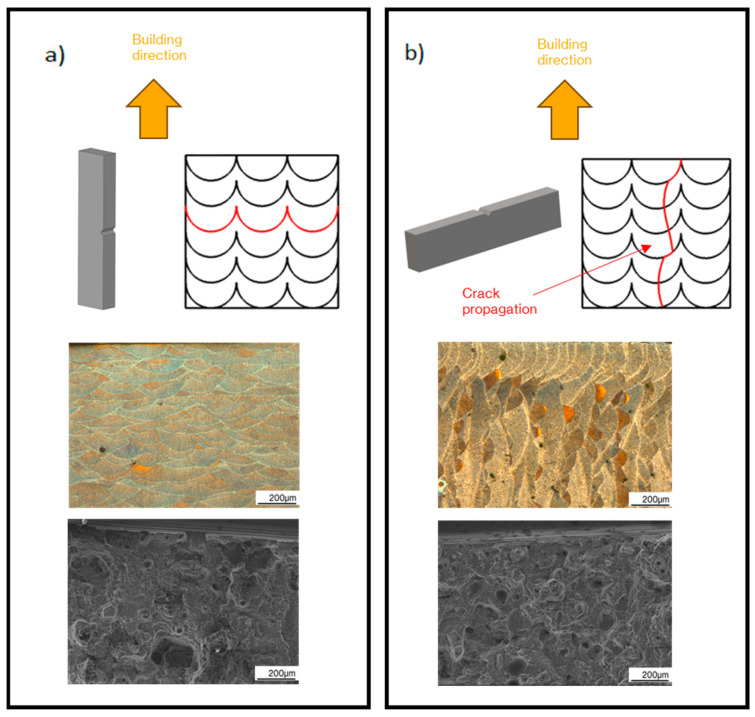
Fracture propagation modes. (**a**) Transgranular fracture, (**b**) Intergranular fracture.

Both crack propagation modes are depicted in Figure 20, where the red line indicates the crack propagation path. As studied by Moses [20], intergranular failure across the grain boundaries dominates in regions where the crack passes the melt pool perpendicularly, whereas transgranular failure is more likely to occur if the crack passes through the melt pool boundaries at an angle. As shown in Figure 20a, where the load application direction coincides with the building direction, the crack propagation is more likely to progress in an intergranular mode, while it progresses in both ways when it does not, as shown in Figure 20b. This is because, in vertical specimens, the crack propagation plane coincides with the direction in which the melt pools are arranged in rows, layer by layer. In this direction, the crack finds a path of aligned melt pool boundaries that oppose an inferior resistance to crack propagation, while in flat and edge samples the crack does not find aligned melt pool boundaries to propagate and must propagate through the melt pools. This explains the difference in fracture toughness between the different building directions, which are more resistant, as the crack must progress to a greater extent through melts pool instead of across them.

Fracture overload regions are shown in Figure 20, together with the microstructure perpendicular to the crack plane for each fracture propagation mode. Certain dimples mixed with cleavage fracture were observed in both samples, which accounts for the elastoplastic fracture behaviour. However, cleave fracture was more pronounced in vertical samples, in contrast to flat and edge samples, where void coalescence is more dominant, which indicates a more elastic behaviour.

## 4. Discussion

The anisotropy of LPBF manufactured samples and the effect of process parameters and border scanning on their mechanical response has been evidenced in this work. Tensile and fracture behaviours of the LPBF AlSi10Mg alloy have already been studied by several authors [16,18,20,38], who obtained different values for strength, stiffness and ductility for each building direction and process parameters, demonstrating the complexity of the LPBF manufacturing process. However, the shear response of the alloy together with the border scanning effect on the different mechanical responses has not been analysed yet and are discussed below.

In this work, vertical samples produced at 60 µm presented a decrease in resistance and ductility of 19% and 64% in comparison with flat 60 µm samples, and vertical 30 µm samples a decrease of 7% and 47% in comparison with flat 30 µm samples (Figure 10). The different surface roughness measured for each layer height may have had an impact on this performance. Samples constructed at 60 µm reported R_a_ and R_z_ values of 25 and 126 µm, whereas 30 µm samples reported values of 9 and 61 µm. Edge samples also showed a decrease in ductility of 37% for both manufacturing parameters in comparison with flat samples due to defects created on the surface because of the supporting structures used, which are too difficult to eliminate and act as stress concentrators causing early fracture. These presented a reduction in resistance of between 6 and 19%.

Residual stresses may have also played a role in the different mechanical responses presented by each building direction. Singh [39] analyzed the residual stress distributions on the different building directions. According to these, vertical samples reported the higher values for tensile residual stresses while flat samples presented the higher compressive residual stress values. The magnitude of stress relaxation in flat samples was also relatively greater in the gauge length section than in the grip section whereas, in vertical samples the magnitude of stress relaxation was primarily localized near the substrate plate. Therefore, each building direction was subjected to a different thermal cycle and presents different values of residual stresses on each zone, which may have also affected the mechanical response [40,41,42].

The increased hardness values obtained for flat samples and the 30 µm process parameters have been related to the higher number of remelted layers present on these configurations (Figure 9), because flat samples, which presented the higher values for hardness, are the only ones in which the indented surface coincides with the top and bottom remelted layers. In addition, the small layer height of the 30 µm process parameters, on which the higher hardness values were found, also reported a higher number of remelted layers. The border scanning also reported an increase in hardness (10 HV) and tensile strength and ductility in all building directions, especially on flat and vertical samples, which reported an increase in energy absorption of 83% and 500%, respectively. Edge samples with border scanning also presented a 43% increase in energy absorption compared to samples without border scanning. Energy absorption increased less in this building direction since the supported zone acted as fracture initiator, as shown in Figure 11.

**Figure 21:**
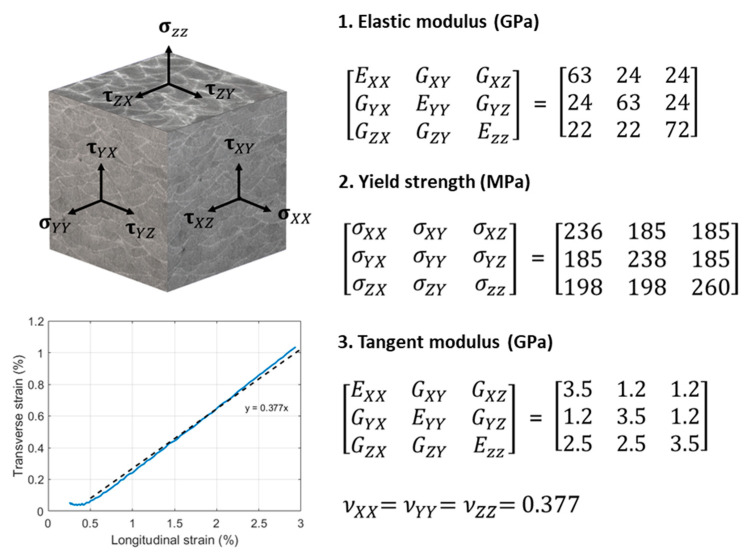
Parameters for the orthotropic material model; dashed line correspond to the interpolation line and the blue line with the experimental results.

An orthotropic material model was built with the elastoplastic properties previously reported, along with the Poisson ratios also obtained in this work, which were similar to those found by [43]. The material model chosen was a bilinear hardening model consisting of elastic and tangent modulus and yield surface, illustrated in Figure 21.

The LPBF AlSi10Mg alloy showed a relatively high mechanical strength and low ductility, due to the fine cellular microstructure produced by the manufacturing process (Figure 8). The shape and size of this microstructure is controlled by manufacturing parameters, since they define the solidification process. The manufacturing parameters used in this work present different values of volumetric energy density (VED), which is a widely used parameter in the literature to compare different LPBF process parameters.

This is because it allows grouping of all process parameters in a single value, which can subsequently be related to the porosity of the samples, giving a measure of the process capacity to produce dense parts. Giovagnoli et al. [26] studied the influence of the VED on the porosity level of the AlSi10Mg alloy produced by LBPF with an EOS M290 system. They obtained inferior values for porosity (1%) for a VED of 43 J/mm^2^ and higher values (4%) for a VED of 66 J/mm^2^. On the other hand, Bertoli et al. [25] demonstrated that the VED is not a parameter which can describe by itself the LPBF process because, in the end, Marangoni flow, hydrodynamic instabilities and recoil pressure dictate the final track morphology. However, it has been reported to be a reliable estimator of the manufacturing conditions. In this work, porosity values of 99% were obtained for VED of 72 J/mm^2^ and 35 J/mm^2^, using the Archimedes method.

The mechanical behaviour of the LPBF manufactured samples is not only affected by the manufacturing parameters but also by the building directions because, depending on them, the melt pools are differently oriented with respect to the load application direction. Their orientation with respect to the load application direction defines the mechanical response of the LPBF manufactured parts, since the stresses must follow different paths to propagate through them, as showed in Figure 20. The border scanning effect on the mechanical response is also attributed to the different arrangement of melt pools in this zone and their internal microstructure.

As shown in Figure 7 and Figure 8, the microstructure of LPBF manufactured samples consist of overlapped melt pools grouped in layers according to the building direction. Furthermore, inside every melt pool there are three different zones: the MPC, the MPB and the HAZ. The difference in resistance between the MPCs and the MPBs explains the different mechanical responses obtained for each building direction. Araujo and Moses [16,20], demonstrated that these zones present a different resistance to crack propagation, where the MPB zones are capable of absorbing less strain energy than MPC zones and are therefore the preferential path for crack propagation.

Similar results were obtained by Patakham et al. [44], who also reported the existence of two types of MPBs, i.e., track–track and layer–layer MPBs. The layer–layer MPBs correspond to the boundaries in which crack propagates through different melt pools, while the track–track MPBs correspond to the MPBs in which the crack propagates through the same melt pool. According to them, the layer–layer MPBs have a more efficient load bearing capacity than the track–track MPBs, which explains the different mechanical responses. Therefore, the higher ductility of flat and edge samples is explained because the layer–layer MPBs are perpendicular to the load application direction in these building directions, whereas on vertical samples they are parallel. In addition, the crack did not have a preferential path to propagate in flat and edge samples, because the MPBs are oriented perpendicular to the crack propagation direction. Nevertheless, in vertical samples, the MPBs are parallel to the crack propagation direction, so the crack encounters a path which propagates along MPBs without having to pass through the MPCs, which requires a higher stress.

**Figure 22:**
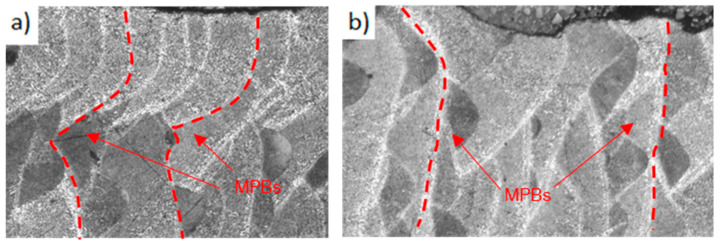
MPBs of edge samples: (**a**) E60. (**b**) E60WB.

The border scanning effect on mechanical properties can be also explained by the different distribution of the MPC and the MPB zones at the border scanned zone. A larger number of MPBs was found on samples with border scanning than on samples without border scanning at the border zone. This increase in MPB zones explains the porosity layer present on samples with border scanning, as pores are easier to form on these zones, as observed in Figure 6 and by Lupi et al. [38]. Although an increase in MPBs has been demonstrated to decrease the mechanical performance [45], the increase in mechanical resistance reported in this work can be attributed to the fact that the high oriented dendrites of the MPB zones faces a discontinuity on the boundary between the core and the border, which hinders crack propagation, increasing the resistance and ductility. Therefore, the different resistances of the layer–layer and track–track MPBs explain the different resistances of both border scanning strategies, as shown in Figure 22.

The long track–track MPBs present at the border scanned zone suddenly face a zone with a major frequency of MPCs from which cracks must propagate or follow the layer–layer MPBs. However, this interaction did not occur under shear stress conditions since, on this case, resistance did not increase. This is because the maximum shear stresses are located at the core of the samples instead of on the external surfaces, which are unaffected by this interaction. The effect of the border scanning strategy has been introduced, but more research is needed on this topic to fully understand the relationship between the microstructure and the mechanical response.

## 5. Conclusions

The impact of building directions, process parameters and border scanning on the mechanical properties of the LPBF manufactured AlSi10Mg alloy has been analysed for different mechanical behaviours. Significant variations in mechanical response have been observed between the different configurations tested, evidencing the effect of these variables on the mechanical response. In addition, an orthotropic material model has been developed with the data obtained, which allows the assessment of the material response prior to its implementation.

Hardness values are reported to be influenced by process parameters, building directions, and the border scanning strategy. Flat samples reported higher values for hardness, reaching values of 150 VH for the 30 µm samples. Both, edge and vertical samples showed inferior hardness values, which were close to 140 HV for the 30 µm process parameters. The samples manufactured with the 30 µm process parameters reported an increase in hardness of around 10 VH in comparison to 60 µm process parameters. Samples without border scanning reported inferior values of hardness (−5 HV) than samples with border scanning.

Building directions reported a significant influence on tensile response, with flat samples showing higher values for strength (400–450 MPa) and ductility (5–6%), followed by edge samples (350–400 MPa, 3–4%) and lastly by vertical samples (350–400 MPa, 1–3%). Process parameters did not show a significant effect on tensile response. In contrast, border scanning incremented strength and ductility, especially on flat and vertical samples, with an increase of around 100 MPa and 1–2% strain. Stiffness was primarily influenced by building directions with vertical samples leaving the higher values, except for without border scanning, which shows the opposite tendency. Yield strength was significantly reduced in these samples, since they did not even reach 200 MPa.

A simple shear behaviour was obtained on shear testing, which proves to be sufficient to evaluate the shear response. Building directions reported an impact on shear strength and strain, with edge samples presenting the higher values for strength (491 MPa) and flat samples the higher values for strain (24%) for the 30 µm process parameters. Flat samples also reported higher values for strain (15%) than edge samples (8%) on the 60 µm process parameters. However, while both building directions reported similar strength values (350–400 MPa) for the 60 µm process parameters, this did not happen for the 30 µm process parameters, where a strength of 350 MPa was obtained for flat samples. Border scanning did not report a significant effect on shear properties.

Building directions were demonstrated to have a higher influence on fracture toughness, where flat samples reported higher values for SIF and j-integral (32 MPa$\sqrt{m}$, 14 kJ/m^2^) followed by edge samples (24 MPa$\sqrt{m}$, 8 kJ/m^2^) and lastly by verticals (19 MPa$\sqrt{m}$, 6.4 kJ/m^2^) for the 60 µm layer height. Process parameters were also proven to have an impact on fracture toughness. Flat specimens built with a layer height of 30 µm reached an inferior SIF (28.6 MPa$\sqrt{m}$) than 60 µm samples, but they absorbed more energy (18.2 kJ/m^2^). The different toughness values were also discriminated by the strain state prior to failure, where a higher plastic zone corresponded to a higher toughness.

In conclusion, the effects of the building directions, process parameters and border scanning on the mechanical response of LPBF AlSi10Mg have been clarified and explained according to its microstructure. Flat samples reported the better mechanical response followed by edge samples and lastly by vertical samples. The 30 µm process parameters reported higher hardness and plasticity for shear and fracture responses and a slightly inferior resistance than 60 µm samples except for shear response. Border scanning reported an increase in tensile mechanical response, whereas it did not have an influence on the shear response.

## Figures and Tables

**Table 1:** LPBF process building parameters.

Conditions Used	Layer Height (µm)	Laser Power (W)	Scanning Speed (m/s)	Volume Hatch (µm)	Energy Density (J/mm^3^)	Build Rate (cm^3^/h)
Infill 30	30	350	1.8	90	72	1.35
Infill 60	60	400	1.6	120	35	3.2
Border 30	30	350	1.2	40	243	-
Border 60	60	350	0.71	50	163	-

**Table 2:** Experimental process.

Testing	Name	Building Direction	Layer Height (µm)	Border Scanning	Samples Tested
Tensile	F60	Flat	60	Yes	3
Tensile	E60	Edge	60	Yes	3
Tensile	V60	Vertical	60	Yes	3
Tensile	F30	Flat	30	Yes	3
Tensile	E30	Edge	30	Yes	3
Tensile	V30	Vertical	30	Yes	3
Tensile	F60WB *	Flat	60	No	4
Tensile	E60WB *	Edge	60	No	4
Tensile	V60WB *	Vertical	60	No	4
Shear	F30	Flat	30	Yes	3
Shear	E30	Edge	30	Yes	3
Shear	F60	Vertical	60	Yes	3
Shear	E60	Flat	60	Yes	3
Shear	F60WB *	Edge	60	No	3
Shear	E60WB *	Vertical	60	No	3
Fracture	F30	Flat	30	Yes	5
Fracture	F60	Edge	60	Yes	5
Fracture	E60	Vertical	60	Yes	5
Fracture	V60	Vertical	60	Yes	5

* WB = Without border.

**Table 3:** Tensile properties of tested samples.

Specimen	Young Modulus (GPa)	Yield Stress (MPa)	Ultimate Strength (MPa)	Strain at Failure (%)	Max. Energy Density (J/cm^3^)
Flat60	63 ± 3	236 ± 6	429 ± 17	5.71 ± 0.5	22
Edge60	63 ± 3	238 ± 5	360 ± 3	3.58 ± 0.13	12
Vertical60	72 ± 6	260 ± 2	344 ± 4	1.80 ± 0.06	12
Flat30	61 ± 5	265 ± 9	425 ± 7	5.20 ± 0.16	20
Edge30	63 ± 3	220 ± 7	399 ± 8	3.20 ± 0.11	9
Vertical30	67 ± 5	206 ± 5	392 ± 15	2.71 ± 0.02	10
Flat60WB	76 ± 4	178 ± 3	347 ± 4	3.98 ± 0.11	12
Edge60WB	68 ± 6	190 ± 3	316 ± 12	2.18 ± 0.32	8
Vertical60WB	54 ± 2	188 ± 4	254 ± 12	0.70 ± 0.07	2

**Table 4:** Shear test results.

Specimen	Shear Modulus (GPa)	Yield Stress (MPa)	Ultimate Strength (MPa)	Strain at Failure (%)	Energy Density (J/cm^3^)
Flat30	25 ± 2	162 ± 1	355 ± 8	24 ± 2	80 ± 7
Edge30	23 ± 3	197 ± 7	491 ± 12	11 ± 1	48 ± 3
Flat60	24 ± 2	185 ± 8	387 ± 8	15 ± 1	45 ± 1
Edge60	22 ± 2	198 ± 2	386 ± 5	9 ± 1	29 ± 2
Flat60WB	21 ± 4	192 ± 7	389 ± 20	15 ± 1	49 ± 1
Edge60WB	20± 4	201 ± 7	350 ± 10	9 ± 2	27 ± 2

## Data Availability

The original contributions presented in the study are included in the article, further inquiries can be directed to the corresponding author.
